# Team interventions in acute hospital contexts: a systematic search of the literature using realist synthesis

**DOI:** 10.1186/s12913-018-3331-3

**Published:** 2018-07-11

**Authors:** U. Cunningham, M. E. Ward, A. De Brún, E. McAuliffe

**Affiliations:** 1 Mater Misericordiae University Hospital, Eccles St, Dublin 7, Ireland; 2 School of Nursing, Midwifery and Health Systems, University College Dublin, Dublin, Ireland

**Keywords:** Team, Interdisciplinary, Interventions, Acute hospital, Effectiveness, Realist, Synthesis, Context, Quality, Safety

## Abstract

**Background:**

Research on team effectiveness in healthcare has focussed on whether effective teams yield positive outcomes for patients and on the effectiveness of team interventions to improve performance. Limited understanding exists of what works for whom within an effective team, or how and why the context in which the team operates enables team members both as individuals and as a collective to enact behaviours that promote positive outcomes.

**Methods:**

This realist synthesis of the literature explores the relationship between team interventions, underlying teamwork mechanisms generated by those interventions, and the resultant impact on patient outcomes in an acute hospital context. A systematic search of five healthcare and healthcare management academic databases: PubMed, PsychINFO, CINAHL, ABInform, Emerald Management and three grey literature databases: ERIC, OpenDOAR and Open Grey was undertaken. Five experts in the field were also contacted to source relevant literature. Using PRISMA guidelines, relevant studies published between January 2006 and January 2017 were systematically searched by a team of three people. Drawing on realist methodology, data were synthesised using context, mechanism and outcome configurations as the unit of analysis to identify enablers and barriers to effective team interventions.

**Results:**

Out of 3347 papers retrieved, 18 were included in the final synthesis. From these, five contextual enablers were identified: an inter-disciplinary focus and flattened hierarchy; effective communication; leadership support and alignment of team goals with organisational goals; credibility of intervention; and appropriate team composition with physician involvement. Ten recurring mechanisms were identified, the most frequently occurring of which was shared responsibility.

**Conclusions:**

The advantage of using realist synthesis to extrapolate data from the literature is that it considers the context and mechanisms that will impact effectiveness of healthcare team interventions. This methodological approach provides a different perspective to other types of syntheses and offers insight as to why certain contextual elements may yield more success than others. Findings therefore tend to have more practical implications. Specificity of detail in terms of how external drivers impact on healthcare team interventions was limited in the articles extracted for analysis. This broader perspective is therefore an important consideration for future research.

**Electronic supplementary material:**

The online version of this article (10.1186/s12913-018-3331-3) contains supplementary material, which is available to authorized users.

## Background

Team performance, quality, safety and efficiency are areas in healthcare that attract a great deal of attention. Given the costs required to operate health services and an increasing evidence base that demonstrates that teamwork failure is a sizeable contributor to patient harm events and preventable medical errors [1–3] this is not surprising.

The extant literature on team effectiveness demonstrates complexity in terms of how healthcare teams are defined. Using previously conceptualised frameworks, Hughes et al. [4] describe healthcare teams as having: low temporal stability, a short team life span and a rotating leadership structure. Schmutz et al’s. [5] portrayal is synonymous with this interpretation making reference to action teams especially those in the dynamic domain of healthcare often working under changing conditions, being assembled on an ad hoc basis and having a dynamically changing team membership. They indicate that they often work together for only a short period of time and consist of members from many different specialties. Gittell et al. [6] and Faraj and Xiao [7] also reflect this dynamic nature pointing out that healthcare teams are synonymous with interdependency and uncertainty. More recently, Edmondson [8] refers to “*teamwork on the fly*” and explores the responsiveness of systems to changing team compositions where members have to co-ordinate and collaborate in the absence of stable team structures.

Healthcare is delivered in teams involving multiple disciplines and the terms multidisciplinary, interdisciplinary and transdisciplinary are often used interchangeably and ambiguously. In their paper, which seeks to clarify this ambiguity, Choi and Pak qualify the three terms, making reference to “the involvement of multiple disciplines to varying degrees on the same continuum” ([9]:225). The common differentiation being as follows: multidisciplinary - additive or staying within their boundaries; interdisciplinary - interactive co-ordinated and a coherent whole; and transdisciplinary - holistic and integrated.

The purpose of multidisciplinary teams in healthcare is usually the delivery of clinical care. However multidisciplinary healthcare teams may also adopt leadership, governance, project management or change management functions. Lemieux-Charles and McGuire reflect on the complexity of healthcare teams in saying “a team is a multi-dimensional construct and team structures and processes can vary widely depending on the membership, work, tasks and interactions”([10]:265). They caution however that studies are frequently lacking in terms of consistency and specificity of detail when describing teams and, as suggested by West and Lyubovnikova, research is required so that we can “accumulate findings” on “real teams” as opposed to “pseudo-like groups” ([11]:332).

For the purpose of this research, we refer to “healthcare teams” as two or more healthcare disciplines working together in an acute hospital context and in receipt of a programme or intervention or directly involved in implementation of a programme or intervention to improve team-working and/or quality and safety of patient care.

Despite the lack of clarity on team definitions, a significant body of literature has emerged on team interventions, team training and team effectiveness as it relates to quality and patient safety and some researchers argue that the relationship of teamwork training to quality of care and patient safety is fundamental.

Over three decades of research on teamwork effectiveness in healthcare, emphasis has been on whether effective teams yield positive outcomes for patients and whether or not team interventions improve team performance. Early studies on team training concluded that there was limited evidence available that linked team training to positive patient outcomes [12]. Subsequently, a systematic review undertaken by Buljac-Samardzic et al. concluded that “only some studies demonstrated high quality evidence on interventions to improve team effectiveness” ([13]:193) and more recently, Weaver, Dy and Rosen found that overall, “moderate-to-high-quality evidence suggests team-training can positively impact healthcare team processes and patient outcomes” ([14]:368).

In her work on learning behaviours in teams, Edmondson [15] explores the factors and contextual conditions that contribute to making teams work more effectively, for example, the impact of leadership behaviours and professional status on psychological safety [16]. From a realist perspective, research is beginning to explore what is it about the resources on offer within an effective team or specific to the context in which the team operates that causes team members both as individuals and as a collective to enact the behaviours or mechanisms that promote positive outcomes [17]. An understanding of these factors could contribute to the design of more effective teamwork interventions as it seems “*how to*” enable and more importantly *“how to*” sustain outcomes of effective team interventions in healthcare still remains a challenge.

Healthcare teams are complex and operate within complex open systems with various healthcare professional groups having their own identity, culture, educational background and objectives. Given the complexity of healthcare teams and the complexity of the healthcare system in which they operate, there are many interacting variables to consider and it is not possible to predict that what works for one team will work for another team and what works in one context will successfully translate to another context.

While there is a large body of research on the importance of context in quality improvement interventions [18–20], there is a gap in the healthcare literature on how these contexts impact on the teams and support or inhibit the enactment of behaviours that are associated with team effectiveness.

As evidenced, many theoretical concepts regarding what constitutes and enables “effective team working” already exist but factors may vary across different settings and different healthcare systems. In order to design successful interventions to improve team effectiveness, it is important to understand the underlying mechanisms that lead to successful outcomes and the factors specific to the contexts that generate these mechanisms.

Realist methodology [21] is tailored to uncover these hidden mechanisms (M) and to elicit the conditions (C) in which they occur and the resultant outcomes (O) they generate. In order for the researcher to extrapolate context, mechanism and outcome configurations C-M-O-Cs, the researcher must make a chain of inference which derives the causal outcome between two events. This requires understanding of the underlying mechanisms that connect them and also the context in which the relationship exists. Patterns of context-mechanism-outcome configurations (CMOCs) across studies or contexts enable the researcher to understand the CMOCs that are common or ‘core’ to an intervention.

For the purpose of this literature synthesis, CMOCs (See also list of abbreviations) are defined as per Table 1.

**Table 1:** Definitions: Context, Mechanism, Outcome Configuration (CMOC)

Context (C)	The conditions in which the programme/intervention is introduced - the enablers/ facilitators/ detractors of teamwork.
Mechanism (M)	The process of how the participant interprets and acts upon the intervention stratagem. How any one of the components of teamwork brings about change. How the resources on offer permeate into the reasoning of team participants.
Outcome (O)	The intended and un-intended consequences of teamwork. Because of the variation in context and mechanisms, there are likely to be different outcomes from teamwork.
Configuration (CMOC)	The patterns and variations in patterns of teamwork*.*

### Aim of systematic search of literature

The aim of this research is to deepen understanding of the relationship between team interventions, underlying teamwork mechanisms generated by those interventions and the resultant impact on patient outcomes in an acute hospital context with the intent of exploring: *What works for whom in what conditions; why, to what extent and how?*

### Specific objectives

Specific objectives of this literature synthesis were as follows:

1. To determine in what conditions and to what extent interventions appear to work best [contexts] (C).
2. To explore how and why team interventions work in these conditions, i.e. what mechanisms (M) are enacted to produce outcomes (O)?

## Methods

Best practice guidelines for the conduct of realist synthesis were followed [22]. Relevant literature on team interventions in a hospital context was interrogated to determine what worked for whom in what conditions, why to what extent and how.

### Searching processes

Five electronic databases relating to healthcare and healthcare management were initially searched: PubMed, PsychINFO, CINAHL, ABInform, Emerald Management. Consistent with best practice guidelines in realist synthesis, three grey literature databases: OpenGrey, OpenDOAR and ERIC were also searched to capture non-indexed studies and grey literature. The following search strategy was used. See Table 2.

**Table 2:** Search strategy, combination of keywords

Search string	Key words
1	Multi-disciplinary team OR Multidisciplinary team OR Trans-disciplinary team OR Transdisciplinary team OR Interdisciplinary team OR Interdisciplinary team OR Inter-professional team OR Interprofessional team OR Patient-Care team OR Patient Care Team OR Patient Facing OR Patient-Facing team
2	Hospital or clinical or medical or health or healthcare
3	1 AND 2
4	Teamwork or dynamics or “team roles” or roles or collaboration or process or processes
5	Group processes
6	Inter-professional relations
7	4 or 5 or 6
8	Effectiveness OR Performance or Efficiency or Experience or Experiences
9	AND 3, 7, 8
10	Limit 2006 - Current

The initial search of databases was conducted in January 2017. Articles that were subsequently published up to July 31st, 2017 were captured via electronic alerts and were considered if relevant. Search of the grey literature databases took place in July 2017. Two senior academics and three national and international practitioners working the field of teamwork in healthcare were invited to contribute recommendations via electronic communication. Bibliographies of relevant articles were also hand searched.

### Narrowing the scope

The initial search addressed the broader question of enablers to effective team-working. This search retrieved 3347 articles relating to team effectiveness of which 819 were scoped by the primary researcher (UC). Preliminary findings were discussed with the research team (EMcA, MW and ADB) and a realist advisory group – this consisted of a group of researchers who were actively using realist methodology and/or academics who had an interest in realist methodology. Studies that involved team interventions appeared to yield the most information in terms of identification of enablers and barriers to team effectiveness. These studies included Lean methodology which focusses on removal of waste to add value for patients and staff. Healthcare organisations use Lean methodologies to streamline processes, reduce cost, and improve quality and timely delivery of products and services. Other team interventions included Quality Improvement, team education, strategies to improve teamwork, implementation of care pathways and introduction of new ways of working or a re-design of existing ways of working. This sub-set of articles also appeared to be evaluated more rigorously in terms of their impact on patient quality and safety outcomes. The following inclusion criteria were therefore agreed.

### Inclusion criteria

Papers on studies published between January 2006 to January 2017 – all languages

1. That were conducted in hospitals and that treated the multidisciplinary team rather than the team member or the organisation as the unit of analysis in terms of team effectiveness.
2. That included a team descriptor - minimum criterion - the type of participants in the team.
3. That discussed all of the following:
  A relationship of a team process variable and clinical or other performance outcome e.g. change or performance improvement initiative.
  Effect on a performance variable through a team process intervention.
  Facilitators and barriers to effective team interventions in healthcare.

All study types were accepted. Studies were excluded if they were deemed unrelated to multidisciplinary teamwork, did not reference the acute hospital context and if there was no reference to team intervention and/or effectiveness. Grey literature was screened for inclusion or exclusion using the same process.

All identified articles were imported into Covidence software [23] to manage the screening process. Papers were independently screened by title and abstract for inclusion or exclusion by two members of the research team (UC, EMcA). Where conflicts arose and were unresolved, a third research team member was invited to make the final decision (MW). Following title and abstract review, the sub-set proceeded to full text review again independently by two members of the research team (UC, MW).

### Selection and appraisal of documents

All documents were appraised for rigour and relevance using the following criteria to explore whether or not:

- they contained data relevant to context that generated outcomes, including unintended outcomes;
- chains of inference could be made in terms of context, mechanism, outcome configurations;
- methods used to generate data were credible and trustworthy.

All studies were selected independently by the primary researcher (UC) in terms of suitability. One of the researchers (MW) rated a random sub-section constituting 20% of the total. As inter-rater reliability was 100%, all studies deemed suitable by the primary researcher were included.

### Data extraction process

In order to identify contextual conditions that enabled or inhibited mechanisms for effective team interventions, information was gathered specific to the type of intervention, the setting in which the intervention occurred, the team description, contextual data on factors that enabled or inhibited effectiveness, mechanisms that were enacted and outcomes of the intervention. The process used for data extraction was significant in terms of its comprehensiveness and data extrapolated were reviewed in terms of their appropriateness by the research team. During the process of data extraction, what constituted a context, mechanism or outcome was discussed in detail. The framework and process for extrapolation of data were also discussed and agreed with members of a realist advisory group. Please see Additional file 1.

### Analysis and synthesis processes

Data analysis and synthesis was initially undertaken by the primary researcher. Using context, mechanism, outcome configurations as the units of analysis, chains of inference of what worked for whom, in what conditions, how and why were made. These were subsequently presented to the research team and realist advisory group respectively in order to ensure consistency and validity of the process.

Patterns of CMOCs that frequently occurred across the 18 studies were identified using the same iterative process. For ease of understanding and practical application, it was agreed that these recurring patterns (referred to in realist terms as “demi-regularities” [22]) would be better represented as plausible hypotheses. These plausible hypotheses are presented below in the form of “if….then” statements. This approach of using plausible hypotheses has previously been employed in realist synthesis research [24–26].

## Results

**Fig. 1:**
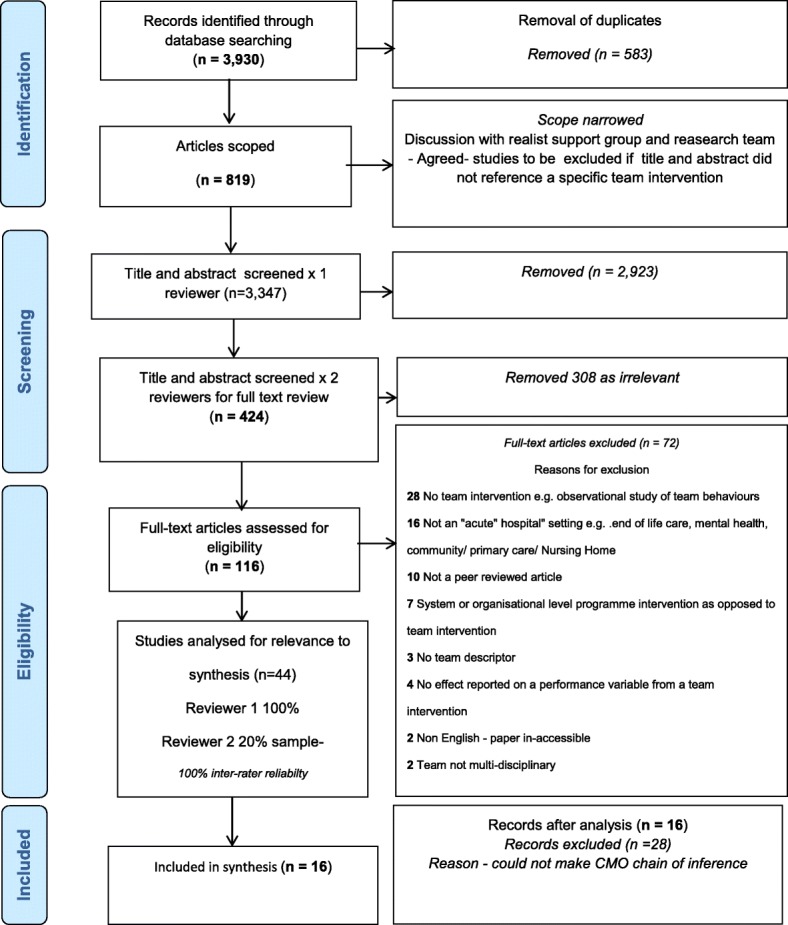
PRISMA flow diagram

A total of sixteen peer-reviewed articles were identified for the synthesis and two additional studies were included from the grey literature searches. No additional studies were added following communication from experts as recommended articles had already been included or excluded based on the criteria. See Fig. 1 and Table 3.

**Table 3:** Grey literature search and document flow

Grey literature Search engine	Search strategy	No. of items screened	No. of items for full text review	No. of items included
ERIC	As per peer review	377	5	1
OpenDOAR	Intitle: teamwork and hospital	First 100 records (results sorted by relevance)	14	1
Open Grey	Team and hospital	86	1	0
Hand searching	Various	0	–	–
Contact Experts	6	Items were already included/excluded	–	–

### Main findings

Within and across the eighteen studies included in the synthesis, five recurring patterns of context mechanism and outcomes (demi-regularities) emerged – many of which are inter-related. These chains of inference are outlined in the form of five “If-then” plausible hypotheses below to explain how contextual factors led to subsequent outcomes via the mechanisms that were generated. Please see Table 4 below and Additional file 1.

**Table 4:** Summary of Findings

	Context	Mechanism	Outcome
	If there is:	this enacts:	and results in:
PH1	Inter-disciplinary focus and Flattened hierarchy	Understanding of roles and Mutual respect, support and value. Shared decision making and common purpose; self and team efficacy.	Increased job satisfaction, higher performance, higher levels of competence, better teamwork and lower feelings of emotional exhaustion. Breaking down of inter-professional silos; more integrated patient care; connectivity of the team and camaraderie.
PH2	Effective Communication: Opportunities for communication; Communication skills; Communication systems	Shared mental models; Clarity of role; Clarity of purpose.	Situational awareness; More integrated care; Better intervention outcomes
PH3	Leadership Support and Alignment of team goals with organisational goals	Motivates, empowers and engages staff, creating a sense of team efficacy and a shared sense of responsibility and accountability.	Team pride; Camaraderie; Connectedness with broader system; Implementation of Intervention; Sustainability of intervention.
PH4	Credibility of intervention provided by experienced trainers who team members can relate to and is perceived to be comprehensive (right amount of core topics) with application to the healthcare context in which the team works	A sense of confidence and engages and motivates team members with the intervention.	High satisfaction; Increased skills, Increased self and team efficacy, Increased role in safety and translation to practice.
PH5	Team composition and Physician involvement consists of appropriately skilled members including a physician, shares a similar foundational knowledge prior to the intervention and participates in a shared learning experience	Shared understanding of the intervention and feel knowledgeable, competent and confident.	Credibility of the intervention, translation to practice and sustainability.

### CMOC 1 Interdisciplinary focus, flattened hierarchy

Plausible Hypothesis 1

**Table:** 

(i) If various healthcare disciplines come together to improve quality of care through a specific intervention, they develop an understanding of each other’s roles and consequently begin to mutually respect, support and value one another, then this results in increased job satisfaction, higher performance, higher levels of competence, better teamwork and lower feelings of emotional exhaustion. (ii) In addition, if there is a flattened hierarchy, then there is shared decision making and common purpose, self and team efficacy resulting in a breaking down of inter-professional silos, more integrated patient care, connectivity of the team and camaraderie.	

This CMOC was strongest in terms of support from the literature. Several authors cite the importance of grounding interventions in interdisciplinary teamwork in order to be effective [12, 27–30].

Interdisciplinary teamwork appears to motivate and empower staff when team members feel their roles and contributions are accepted, valued and acknowledged across the organisation. For example, implementation of an interdisciplinary team-based approach led a tracheostomy care team to reflect that:

“The team feel their role and contribution are accepted, valued and acknowledged across the organisation. Some commented that they felt their professional profiles and that of their discipline were enhanced as a result of their participation in the team and in the implementation project. They also reported feeling respected and valued by other team members” ([28]:1281).

In this instance, new awareness of each other’s roles and contributions resulted from the formation of the team and resulted in their ability to work to complement each other’s inputs.

Similarly, for one quality improvement team coming together as an interdisciplinary team in order to contribute to Plan, Do, Study, Act (PDSA) cycles also proved effective and enacted mutual support respect and value with significant positive outputs for patients.

The success was explained as follows:

“A PDSA process requires commitment at all levels over the long-term. Individual team members must support the team, for example, by arriving on time to the OR, making sure case posting and orders are accurate, communicating effectively, and honestly evaluating their responsibility and contribution to teamwork and team function” ([29]:342).

The benefits of different professionals concurrently reviewing individual results for example was explained by Riblet et al. [31] as enabling team members to see how their work connects to others, and leads to shared decision making. In this specific context, this type of collaborative effort fostered camaraderie and motivated team members to remain engaged in the work despite obstacles.

The evidence to support interdisciplinary teamwork is therefore strong. Importantly, the type of hierarchical structure however appears to moderate the degree of effectiveness of interdisciplinary teamwork - a flattened hierarchy leading to a stronger team identity and feelings of increased satisfaction and collegiality, enhancement of patient comfort and improved standards of care [12, 28, 32].

Such a system “fosters teamwork and breaks down silos” and “members feel good about team membership”([32]:1281). In these instances it appears that self and team efficacy was enacted. In contrast, the tradition of “surgeon primacy” appeared to negatively impact self and team efficacy. Professional power, status differences and inter-professional tensions within the traditional medical hierarchy were identified as potential barriers to inter-disciplinary team identity and connectedness [31–34]

### CMOC 2 Effective communication

Plausible hypothesis 2

**Table:** 

If team members communicate effectively, then this leads to increased situational awareness, more integrated care and/or better intervention outcomes because team members feel there is clarity of purpose and role and they share mental models with other team members.	

Team success is contingent on effective communication and this was the most frequently cited contextual enabler for team interventions and team effectiveness [5, 27–29, 32, 34–37].

Enablers relating to *effective communication* are differentiated into three sub-groups: opportunities for communication and transfer of knowledge; communication skills; and communication systems. This was important in order to understand if mechanisms enacted were specific to each.

#### Opportunities for communication

A number of opportunities for teams to come together for knowledge transfer are described in the literature. They include team rounding, team meetings, QI interventions, development of care pathways, PDSA cycling etc. These collaborations allow opportunities for team members to learn from one another and about one another. Parker et al. demonstrate the benefits of a new interdisciplinary tracheostomy team round in terms of allowing for more effective communication between team members and thereby increasing awareness of each other’s involvement. Reflecting on the process, they found:

“In contrast to their previous experience of an ‘isolated, ad hoc’ approach to communication and co-ordination, they are now more aware of each other’s roles and contributions and can work to complement each other’s inputs. They expressed the view that the team approach has led to greater patient comfort and improved standards of care” ([28]:1281).

In a study to reduce the rate of occurrence of pressure ulcers, Donovan et al. describe interdisciplinary ward rounding and use of integrated documentation and interdisciplinary rounding checklists as antecedents to situational awareness.

“By discussing risk and skin integrity during interdisciplinary rounds and by having patient skin condition documented in a single location, our initiative ensured that all providers were aware of the status of patients” ([38]:46).

Similarly, the process of the team coming together for QI interventions, for example, development of care pathways, helped to reduce role ambiguity among workers and standardised delivery of care by clarifying roles where there is high task uncertainty [33]. Evidence from this study suggests that during the process of co-developing the care pathway, team goals are defined, a team vision is built and concerns are shared on quality of task performance and task orientation.

#### Communication skills

A number of studies emphasised the importance of using different communication skills in specific contexts: for example use of the ‘Red rule’ to allow team members to speak up and voice concerns in the operating room (OR) [35]; use of assertive language and voicing of concerns in the emergency department (ED), [32] and use of closed loop communication in intensive care units and EDs [5].

Training in communication skills also appeared to lead to improvements in situational awareness. Cima et al. referring to communication skills training, stated:

“This protracted effort, which stressed improving direct communication between all team members to improve OR situational awareness has been shown to be one of the most important components for successful institutional change” ([35]:129).

Following the intervention, the practical use of a white board as a communication system served as a method to standardise reporting of items used and any items that were removed, thus improving the awareness of items placed in the surgical field.

As a corollary, the lack of structured communication systems was cited as an inhibitor to shared decision making and less-than-ideal process performance. In their study to improve care of patients with glioma, Riblet et al. [31] indicated that prior to the intervention, staff had to make decisions based on a narrow set of variables because there was no system to facilitate communication among services. There was also a lack of awareness of each other’s contributions, which impacted on the team’s ability to deliver integrated care.

Evidence suggested that other contextual variables in complex, high pressured and stressful situations can also moderate the ability to communicate effectively and impact transfer of knowledge which in turn can impact negatively on situational awareness - Schmutz et al., cite Bandow (2001:

“In an emergency task, factors like time pressure, noise (e.g., from the vital signs monitor), and simultaneous talking may result in misunderstandings or even lack of understanding among team members. Furthermore, individuals can receive different messages when hearing the same communication due to personal biases and perspectives” ([5]:763).

#### Visual management systems

Use of visual management systems for performance monitoring was particularly effective in intervention studies that used QI and lean methodology [27, 39].

Developing data dashboards that were periodically reviewed with team members were important in helping to engage in dialogue to review performance and to sustain project gains. They served to maintain clarity of purpose as well as helping to identify new areas for improvement [37].

The location of these was seen as being significant and an inhibitor in one study where the PDSA team found that posting routines and communication between staff members at the surgeon’s office and the OR scheduling desk were primary problem areas [29]. Equal ownership and interaction with the visual management systems by all team members required that the data was posted in a space that was easily and equally accessible by all team members.

### CMOC 3 Leadership support, alignment of team goals with organisational goals

Plausible hypothesis 3

**Table:** 

If there is leadership support for interventions and team goals are aligned with organisational goals, then this motivates, empowers and engages staff, creating a sense of team efficacy and a shared sense of responsibility and accountability. This results in team pride, camaraderie, connectedness with the broader system and is more likely to result in implementation and sustainability of the intervention.	

This CMOC was particularly evident in one study which involved the introduction of the evidence-based Team Strategies and Tools to Enhance Performance and Patient Safety (TeamSTEPPS) training programme.

In their paper, Thomas and Galla, describe the positive impact of leadership support for the TeamSTEPPS intervention process which was formalised via a structured council structure and served to empower and engage team members when implementing the programme:

“Executive leadership at the pilot hospital played a crucial role in sustainment, choosing to continue as a team beyond implementation, and have evolved in their role and functions; the team continues to provide oversight and facilitate TeamSTEPPS, empowering and engaging staff through the council structure and contributing to sustainment of TeamSTEPPS” ([30]:428).

In one study [31], use of dashboards to share performance data allowed for on-going dialogue between frontline staff and management and enacted a sense of collective responsibility. In contrast, prior to the intervention, participants:

“Had limited awareness of the extent to which individual steps contributed to the larger system of care. There was no mechanism in place to facilitate on-going process feedback” ([31]:149).

Provision of practical support in the form of dedicated time for critical training in two studies demonstrated commitment of the organisation and was therefore motivating. [30, 32] Being held accountable by leadership for performance was also perceived positively and served to engage and motivate staff towards successful outcomes [27, 31]. Where the role or purpose was clearly defined and simultaneously linked to organisational strategy, this seemed to be particularly effective.

In one study to improve immunisation rates, prior to project implementation “the team identified the projects as aligning with the hospital’s strategic initiatives”([27]:311). It was important for frontline staff who owned the process to have leadership support and this study demonstrated that aligning educational objectives with the hospital’s strategic initiatives could lead to positive educational outcomes and more efficient care delivery through teamwork with faculty, residents, and hospital staff.

Rosen et al., [12] also point to the importance of alignment of team interventions to strategic goals as this appeared to reinforce the purpose of the intervention and was therefore motivating for staff. The effectiveness of communicating the relevance and permanence of a new way of working and how team goals align with the organisation’s quality and safety goals is described by Thomas and Galla:

“The importance and relevance of TeamSTEPPS was communicated to everyone at the start of training by communicating its connection to the organisational vision and mission via the collaborative Care Model. The message was that TeamSTEPPS was now the way we would conduct business” ([30]:428).

In addition, recognition and acknowledgement by leadership of how the team had contributed to the broader organisation via a show case event was an important enabler in fostering a feeling of pride among team members and on-going team development. Referencing an annual site wide poster session, the authors noted:

“The enthusiasm was contagious, and there was tremendous pride in displaying their achievements. This camaraderie and sharing enabled further team cohesion and learning” ([30]:429).

### CMOC 4 Credibility of intervention

Plausible hypothesis 4

**Table:** 

If the intervention is provided by experienced trainers who team members can relate to and is perceived to be comprehensive (right amount of core topics) with application to the healthcare context in which the team works, then this enacts a sense of confidence and engages and motivates team members with the intervention resulting in high satisfaction, increased skills, increased self and team efficacy, increased role in safety and translation to practice.	

Perceptions of staff with regard to the quality of training or intervention appeared to impact on their satisfaction level and application to practice afterwards. Evidence of staff satisfaction being high corresponded to perceptions of training being of high utility. This was rationalised by Chiccochio [40] in saying that it is essential for healthcare professionals to feel that time away from patient care is well invested. Mayer et al. [41] also acknowledged this requirement for success and designed their intervention specifically to decrease staff time away from bedside.

Success of simulation events is attributed in the main to their clinical relevance and immediate application to practice. To ensure clinical relevance for participants and to optimise authenticity, Figuero, Sepanski and Goldberg [37] ensured that cases used in training were based on real events that occurred in the team’s environment. This resulted in increased confidence levels in the programme. As well as improving technical skills, this in turn led to increased confidence in ability to translate new skills to practice during crisis scenarios and confidence to lead a future resuscitation event.

Simulation delivers successful outcomes in terms of improvement of technical skills and awareness of safety issues. According to Patterson [32] simulation-based learning, specifically when accompanied with video assisted debriefing, allows for team members to experience the event and reflect. Using video assisted debriefing makes the potential to harm patients very real to the participants and this authenticity appears to be what motivates and engages staff.

“The emotional and behavioural engagement engendered by participation in simulation-based teamwork has the potential to change the participant’s beliefs about the individual’s and team’s roles in patient safety and thereby lead to culture change within the microsystem” ([32]:391).

The simulation intervention and the reflection associated with it promoted connection with the actual clinical environment. In these scenarios, the importance of team functioning and cross-checking is brought to the fore in a way that traditional classroom training cannot match. Continuous practice of skills to reinforce the desired behaviours in “near enough” real situations also increased staff confidence ([32]:5).

For service improvement interventions, such as QI or Lean projects, the explicit nature of the process and specificity of goals appears to be the factor that engaged and motivated staff and increased confidence [27, 29]. In these interventions, a systematic approach to improvement was employed that allowed for structured learning experiences and opportunities to clarify expectations. This helped to increase the team members’ understanding of the new process and their feelings of confidence which resulted in successful outcomes.

Nakayama describes the following:

“At the end of the elective day, once per week, the walkthrough scenarios provided an opportunity for team members to practice procedures such as an emergency tracheotomy and malignant hyperthermia. These interactions allowed team members to clarify expectations for their actions during urgent circumstances” ([29]:338).

In these studies, post intervention performance was also closely monitored. For example, in the case of the Lean intervention study, team performance was graded per procedure and in the QI study everyone was held accountable through weekly team meetings and monthly reporting sessions. This also helped to maintain staff engagement and motivation. In terms of outcome, there was high satisfaction with this type of structured learning experience. The didactic and experiential learning appeared to be

“powerfully synergistic, and the patient care improvements were motivating to the teams” ([27]:317).

Thomas and Gala [30] provide further evidence that healthcare teams are more likely to have confidence and engage in programmes if they feel they are credible. For instance, despite TeamSTEPPS being an evidence-based intervention, healthcare staff from various teams only deemed it valid when there was evidence of results:

“There was concurrence on wanting this new reality of a transformed culture; however, the prevailing attitude was one of believing in TeamSTEPPS utility only when the desired changes in environment were observed; emotions ranged from excitement to scepticism” ([30]:428).

This credibility factor also appears to be dependent on the use of experienced trainers that staff could relate to. This encouraged the engagement of participants in programmes or interventions and was therefore an enabler to effectiveness. The use of skilled trainers was highlighted in a number of studies: Riblet et al., [31] referenced the importance of having a coach trained in QI methodology; Hina Syeda [27] employed a QI instructor and Master Black Belt; while Mayer et al., [41] indicated that one of the key success factors for the programme was the strong physician and nurse leaders who had previous experience in organisational change.

Thomas and Gala were clear in stating the need for physicians to be involved in training their colleagues:

“Physicians must be engaged as champions who believe in the importance and value of the training” ([30]:433).

Their experience demonstrated that physicians responded best to training conducted by other physicians and therefore it was important that physicians were seen as leaders in TeamSTEPPS training and rollout.

The literature describes a number of interventions that resulted from a national policy for example TeamSTEPPS, Adoption in Action [41], or a Sentinel Alert [39]. It is possible that this could have contributed to the credibility of the intervention, however detail is not provided and therefore inferences could not be made.

### CMOC 5 Composition of team and physician involvement

Plausible hypothesis 5

**Table:** 

If the team consists of appropriately skilled members including a physician, shares a similar foundational knowledge prior to the intervention and participates in a shared learning experience, then team members will have a shared understanding of the intervention and feel knowledgeable, competent and confident resulting in credibility of the intervention, translation to practice and sustainability.	

The composition of the team implementing the intervention or undertaking the training programme is key to success. This appears obvious as per Ellaham:

“getting the right people involved is important” and “when the multidisciplinary team is involved, this assists in problem identification, and makes changes easier.” ([42]:3)

In a number of interventions, team members were purposively selected to ensure the team was composed of the right knowledge, skills and attributes. Nakayama [29] describes and intervention to improve the surgical service line:

“The PDSA team identified anaesthesiologists, nurse anaesthetists, nurses, and surgical technologists who had the necessary certifications, skills, experience, and interest in paediatric surgical procedures to unite as the paediatric OR team” ([33]:341).

Deneckere also supports this idea and adds that this will happen naturally when developing care pathways:

“The team will define the most appropriate team composition and an inter-professional team with complementary skills (perceived teamness) will be built.” ([33]: 101)

Very strong evidence emerged to support physician involvement in interventions as this was considered fundamental to success of the intervention [5, 27, 30].

Authors attributed successful outcomes to physician involvement where physicians led the intervention or were actively engaged in training and/or training others [30]. Translation to practice was strengthened when coupled with nurse leadership involvement at unit level. For example, as part of an intervention to reduce risks in the labour and delivery suite, Shea Lewis [39] describes changes in the process used to track calls from physicians and changes made to the documentation by nursing staff regarding location of covering physicians. This resulted in increasing nursing awareness of the physicians’ plan for every patient before they were admitted to the labour and delivery suite. It was also the catalyst for nurse education to engage with the department of anaesthesia for in-service training reviewing the role of nursing staff assisting with general anaesthesia.

The importance of starting training from a similar knowledge base is identified as a necessary foundation on which to build skills.

“Some facilitators from both simulation centres felt that depth and understanding from debrief discussions were substantially determined by participants’ starting points.*”* ([34]:102)

Sharing a similar experience of team training is also important for successful translation of skills learned into practice [31, 32, 39, 41]. In simulation or TeamSTEPPS programmes, issues arose when this did not occur [30, 32]. For example, if staff were rostered to work with other staff who did not participate in the respective programme, it was difficult to implement learning. For this reason, Patterson advocated for “mandatory participation of all ED staff in the programme” ([32]:384) which addressed safety issues in the ED explaining that this ensured that all personnel had a similar foundation concerning behavioural expectations and attitudes related to communication and team behaviours. Thomas and Galla [30] also support the training of all staff and in addition advocated the review of all competencies on an annual basis to increase likelihood of sustainment of teamwork behaviours promoted through TeamSTEPPS implementation.

Given the high turnover in healthcare, “team churn” was identified as a potential barrier to sustainability [40]. However, in the same study, the authors reference the fact that the on-going training programme employed in their organisation might help to overcome this:

“Although it is suspected that member churn affects team training performance, the more substantive question is how teams adjust to composition changes with regard to retaining knowledge that leaves the team on the one hand and transferring knowledge to incoming members on the other hand as the new knowledge is being acquired in longitudinal training” ([40]:29).

Despite what was deemed appropriate team selection in terms of knowledge and skills, a moderator of team success in implementing the intervention was the entrenched hierarchies and inter-professional power inherent in some teams. As cited by Cima, this led to:

“…a culture lacking in basic communication skills, poor situational awareness, and concern about questioning the course of events in the OR” ([35]: 130-131).

This barrier was overcome in Patterson’s study where:

“The intervention included training in respectful assertion and voicing of concerns (deference to expertise, advocacy and enquiry) and closed loop communication as well as opportunities for less powerful team members to practice these skills in a simulated setting.” ([32]:389)

## Discussion

This review of the literature considered the topic of effectiveness of team interventions in healthcare using realist synthesis. It therefore adds to the existing literature including a recent narrative synthesis of team training [14]. Realist methodology lends itself well to the review of team interventions accounting for context, mechanisms and outcomes in the process of systematically and transparently synthesising the relevant literature [43]. Findings provide nuanced insight into the mechanisms enacted in various contextual conditions associated with team interventions and how and why particular contexts generate outcomes, including unintended outcomes and have the potential for more pragmatic conclusions than alternative approaches to literature reviews [44].

Building on a previous realist synthesis by Hewitt, Sims and Harris [17, 44–46] which identified thirteen different mechanisms underpinning inter-professional teamwork in health and social care, this study deepens understanding of underlying teamwork mechanisms when team interventions are introduced to or by multidisciplinary teams in acute hospital settings. By analysing interconnectedness between context, mechanism and outcomes and also inter-dependencies across contexts, important detail relating to team interventions emerged.

Five frequently recurring patterns of contexts, mechanisms and outcomes have been presented as “*plausible hypotheses” (PH)*. Not surprisingly, effective communication was the most frequently cited contextual enabler *(PH2)*. This is important as where teams communicate more effectively, they share mental models, perform more teamwork behaviour, and thus perform better [5].

Effective communication and tactical communication were identified by Hewitt et al., [17, 46] as underlying mechanisms for successful inter-professional teamwork. In our findings, we identify effective communication *(PH2)* as a contextual enabler for team interventions and differentiate between three different contextual variables therein. These include: opportunities for communication and transfer of knowledge; communication skills and communication systems. The detailed level of analysis from a realist perspective provides insight as to how and why these are important enablers by unpacking the mechanisms generated by the conditions. Rather than the more generic statement that effective communication is important, this type of analysis allows an understanding of how and why: because team members as individuals and a collective have clarity of purpose and role and share mental models resulting in increased situational awareness and more integrated care. The methodology also allows for further exploration of contextual moderators for example, in one instance, despite team training in specific communication techniques being provided, environmental noise in ED was a significant moderator in terms of success of the intervention. Without this more detailed analysis of context, mechanisms and outcomes generated, valuable information for implementation of interventions could be lost.

The use of evidence based programmes and experienced trainers were critical to success (*PH4*) and staff ability to relate to the programme providers appeared to be equally important [39]. Using physicians and nurses who are experienced in organisational change that staff could relate to on a clinical level therefore appeared to be particularly effective. Thus, credibility of intervention *(PH4)* was extrapolated as a key enabler. Going deeper into the reasoning of team members to the resources provided by credible interventions *(PH4)*, the motivating influence appears to be the authenticity and specificity of the intervention that enacts confidence and team satisfaction, and these are necessary because healthcare workers need to feel time away from patient care is well spent.

In keeping with Weaver, Dy and Rosen’s [14] findings, evidence from the empirical data was also strong to suggest an interdisciplinary approach to training as being essential to the acceptance and commitment of the teams for the specific team intervention *(PH 1)*. However, a consideration upon further exploration is that it is through the interdisciplinary approach that hierarchical issues emerge and importantly therefore it is the coupling of an interdisciplinary approach with a flattened hierarchy that is the enabling condition.

The importance of the inter-dependency of contextual conditions is also evident in *PH3 - Leadership support and strategic alignment of team goals with organisational goals*. As illustrated in one study, [30] joint review of performance via the use of dashboards provided a vehicle for senior management to interact with the team and it was this engagement with leadership that made explicit the team’s role in the overall organisational strategy and enacted a sense of shared or collective responsibility. If analysis treated leadership support and strategic alignment of team goals with organisational goals as two discrete contextual enablers, this important detail would have been lost.

Inclusion of staff with the appropriate skills, knowledge and attributes was also deemed important (*PH5*) and inclusion of physicians in training and delivery is particularly powerful as it provides varied perspectives to the learning and discussion and demonstrates visible organisational commitment *(PH3)* to participants. As with PH1 and PH5*,* interconnectivity between physician engagement and team composition needs to be acknowledged. Broad team composition is only an enabler if there is physician involvement and physician involvement is a necessary condition that probably would not work without broad team composition.

These plausible hypotheses have practical application for teamwork interventions in acute hospital contexts. Within this context however, certain types of interventions have more relevance. For example, because emergency situations are highly stressful, it is not enough to train team co-ordination behaviours just once. Stressful situations can reduce cognitive functioning and thus it is easier to rely on well-learned, automated behaviour. It is necessary to train emergency situations regularly and integrate them into everyday work practices [47].

Weaver et al. [14] describe the need for bundled interventions and there was evidence to support this in a number of studies for example the integration of TeamSTEPPS with Collaborative Care Councils [30]. Schmutz et al. [5] also advocate for bundling closed loop communication skills training with teamwork training. In these circumstances, simulation programmes are usually considered to yield positive outputs. Simulation may however also be appropriate to other healthcare contexts as the use of video assisted briefing and de-briefing could be modified in such a way that it incorporates real life situations from any of these contexts. Given that communication skills are the most frequent skill being analysed and improved, it is possible that simulation could also translate well to any non-pressurised, non-complex care area.

The strength of the synthesis in comparison to other literature reviews is that rather than explore team training or intervention content and effectiveness [14, 48], it allowed for consideration of the contexts and mechanisms that may have operated as enablers or barriers in implementation efforts and highlighted contexts that are most likely to enact team mechanisms leading to positive team behaviours, and as a consequence the quality and safety of patient care.

Weaver et al. [14] concluded that there is moderate to high quality evidence that team training works but do not give important contextual detail as to why this happened. This review of the literature using realist synthesis offers a rationale via the identification of mechanisms enacted by those contexts as to why certain contextual elements may yield more success than others beyond simply the components of the simulation, Lean, QI or service improvement initiative. This contextual data is important for replication of interventions in other contexts as it can help predict why an intervention might fail or flourish and help recommend why certain conditions need to change in advance of implementation in order to optimise chances of intervention success. By way of example, it is worth considering and addressing an interprofessional hierarchy and/or power struggle within a team prior to implementing an intervention as interprofessional tensions may impact negatively and detract from its success. Similarly, a first point to start working with improving communication in teams would be to ensure they have a shared understanding of their vision, mission and goal.

One challenge encountered during the research was the lack of detailed information with regard to teamwork mechanisms and contextual factors that drive success in the literature. Despite a high number of potential studies on this topic being retrieved from databases searches, only 18 reached the final synthesis. Several intervention studies focussed heavily on discussion of the specifics of intervention components [49] or aspects of an interventional model [50]. Many studies considered a process change that incorporated Lean methodologies [51] with little or no reference to the team processes or behaviours involved in changes in their discussion of findings. Thus, this limited the data available for synthesis purposes. Given this, we strongly advocate for adherence to intervention reporting guidelines (TIDieR) [52] to enhance the description and replicability of interventions, and ensure inclusion of relevant information on contextual variables of the team intervention *(who, where and what)* and on the theory or mechanisms underpinning the intervention *(how and why).*

Studies included in the synthesis all demonstrated positive outcomes in terms of performance improvement following the team intervention. This occurred despite criteria allowing for studies that identified barriers to teamwork with negative outcomes. This lack of reporting on negative outcomes could be reflective of what researchers seek to publish or journal publication bias. Arguably, both are unlikely to publish studies with more negative results.

The literature describes a number of interventions that resulted from a national policy for example TeamSTEPPS, Adoption in Action [41], or a Sentinel Alert [39]. This driver for implementation could have impacted on its success. The influence of a national policy or drive has not been considered in this synthesis as the unit of analysis was at the team level and there was vagueness in these studies about the specifics of these policies and how they might have impacted on the team intervention. It was therefore difficult to infer rationale for the impact. This factor is worth exploring further in future research.

## Conclusions

Although complex, realist synthesis allowed for unique insights into mechanisms generated by various contextual conditions. Previous studies largely focussed on whether interventions worked or not, this study examines in more depth the *how and why* they work or do not work. Chains of inference do not occur as discrete entities, rather they are interdependent and exploration of these inter-dependencies yields valuable information. Attempts to over-simply contextual conditions could result in loss of critical information. On a cautionary note, it cannot be assumed that because a particular contextual condition appears to lead to a positive outcome, as a corollary that the absence of that condition will imply a negative outcome. The chains of inference are better served if interpreted as acting along a continuum and inter-dependently.

The five plausible hypotheses have a practical application; however, some types of interventions are more suited to particular contexts than others.

## Additional file

Additional file 1: **Hyperlink 1**. Data extrapolation template for studies included in synthesis. **Hyperlink 2.** Teamwork mechanisms identified. (DOCX 70 kb)

## Abbreviations

CMOC: Context, mechanism, outcome, configuration
ED: Emergency department
ICU: Intensive care unit
OR: Operating room
PDSA: Plan, Do, Study, Act
QI: Quality improvement
RAMESES: Realist and Meta-narrative Evidence, Evolving Standards
TeamSTEPPS: Team strategies & tools to enhance performance and patient safety
