# Supplementary material for: Team interventions in acute hospital contexts: a systematic search of the literature using realist synthesis
Source: BMC Health Serv Res. 2018 Jul 11;18:536. doi: 10.1186/s12913-018-3331-3 (PMC6042358; doi:10.1186/s12913-018-3331-3)
Supplement: Supplementary file 1 — Hyperlink 1. Data extrapolation template for studies included in synthesis. Hyperlink 2. Teamwork mechanisms identified. (DOCX 70 kb) [file 12913_2018_3331_MOESM1_ESM.docx]

**Additional file 1**

**Hyperlink 1 – Data extrapolation templatefor 18 studies included**

| **Cima et al., (2009)** | Reducing RFOs | Tertiary referral centre | Leadership team- Surgeons, Nurses, QI personnel, Administrative staff, sentinel event team members | Intervention led by team over three phases, defect analysis and policy review, awareness and communication, control and monitoring | Multi-centre, multi-phase interventional | Support, Situational awareness, Shared understanding, Shared mental models | Use of red Rule to allow teams members to speak up | Average interval for RFO occurrence increased from 16 days pre-intervention to 69 days post intervention |  |
| --- | --- | --- | --- | --- | --- | --- | --- | --- | --- |
| **Chiccochio (2015)** | Collaboration and project management training | Multi-centre University Hospital | 14 project teams in five locations:, Physicians, Nurses, Pharmacists, Nutritionists, Support Staff | Task work and teamwork training over three half day workshops | Hypothesis driven quality evaluation | Clarity of role & purpose Connectedness, Camaraderie, Teamness, group identity Feeling knowledgeable, feeling competent | Comprehensive team training Specific to needs, Just in time Relatedness of trainers | High satisfaction and usefulness of course Improved self-efficacy Increased goal clarity and co-ordination, improvement in functional performance of projects |  |
| **Deneckere et al., (2013)** | Implementation of Care pathways (CPs) | 39 acute hospitals in Dutch, Clinical pathway network | COPD and orthopaedic teams including surgeons, pneumonologists, Nurses, physiotherapists and social workers | Implementation of CP | Clustered RCT | Mutual support, respect and value, Sharing responsibility, collective responsibility, Connectedness, Camaraderie, Teamness, Group identity | Identification of team goals, IP relationships, Fragmented organisational structures, Matrix management Professional boundaries, status, power, LSS Structured Team based process. | CPs relationship with teamwork seems to be primarily triggered though their ability to improve team level task work. Post intervention. Better perception of teamness, Lower exhaustion and higher level of competence perceived by those who implemented CP |  |
|  |  |  |  |  |  |  |  |  |  |
| **Author (Year)** | **Brief name of study** | **Location** | **Team descriptor** | **Intervention details** | **Study methodology** | **Teamwork**  **Mechanisms described** | **Contextual factors** | **Findings/Outcomes** |  |
|  |  |  |  |  |  |  | Structured methodology |  |  |
| **Donovan et al., (2016)** | Reducing unit acquired pressure ulcers (UAPU) | Tertiary referral centre | Nursing leadership, Nursing specialists, support staff, nutritionists, physical and occupational therapists, physicians, researchers | Lean Six Sigma | Process improvement Lean methodology | Situational awareness, shared understanding, shared mental models |  | UAPU rate reduced from 4.45 to 2.9% during and post intervention in control phase. |  |
|  |  |  |  |  |  |  |  |  |  |
| **Ellahham et al., (2015)** | Door to balloon time reduction for STEMI patients | Tertiary Referral Centre | Cardiologists, Catheterisation laboratory personnel, ED caregivers and Quality staff | Lean Six gma | Process improvement lean methodology | Clarity of role or purpose  Shared / Collective responsibility | Right people involved Communication to all areas. DMAIC cycle, Simple process, Leadership and management support, Empowerment of frontline staff | Decrease in door to balloon time from 75.1 minutes to 60.1,% of patients receiving PCI within 90 minutes increased form 93% to 96%Failed to provide evidence of better patient outcomes |  |
|  |  |  |  |  |  |  |  |  |  |
|  |  |  |  |  |  |  |  |  |  |
|  |  |  |  |  |  |  |  |  |  |
| **Author (Year)** | **Brief name of study** | **Location** | **Team descriptor** | **Intervention details** | **Study methodology** | **Teamwork**  **Mechanisms described** | **Contextual factors** | **Findings/Outcomes** |  |
| **Figuero, Sepanski and Goldberg, (2013)** | Post paediatric cardiac surgery arrest simulation based training | PCICU simulation centre | 37 participants, Nurses, cardiology, critical care trainees, respiratory therapists and 4 non categorised | Off-site 9 hour simulation course | Hypothesis driven evaluation | Feeling knowledgeable and competent, Sense of empowerment and confidence | Distribution of tasks, Evidence based Team Stepps, Use of algorithm, Clinically relevant, Specific goals and objectives, Aligned with clinical practices | Increased Confidence level Closed loop communication, use of huddles, de-briefing, perception of mutual respect, Sense of empowerment. |  |
| **Hina Sayeda, (2013)** | Pneumonia and influenza immunisation | Tertiary Referral centre | Leadership team, Chief medical officer, institutional official, quality Director, residency programme director, Project team ,Resident champions, Nurse management, IT staff, Quality co-ordinator | Structured curriculum for QI and teamwork for MDTs,5 days organised training sessions | Lean Six Sigma | Motivating, Satisfying, Engaging, empowering, Clarity of purpose, Shared accountability, responsibility, commitment, Valued contribution, self and team efficacy, Feeling knowledgeable and competent | Resident involvement, Alignment with organisational goals Critical education team training, Weekly team meetings and monthly reporting, Working in inter-professional teams, Experienced trainers | Positive learning outcomes and more and improvement in vaccination rates z score of 1.96 increased to 3.9 |  |
|  |  |  |  |  |  |  |  |  |  |
|  |  |  |  |  |  |  |  |  |  |
| **Mayer et al., (2011)** | Implementation of TeamStepps | Tertiary referral centre, paediatric and surgical ICU | Nurses, physicians, Respiratory Therapist, Patient Safety Officer | TeamStepps implementation | Interventional evaluation study | Mutual respect, Support and Value, Clarity of role, Sense of accountability and responsibility | Master training, Bespoke programme Alignment with organisational goals, Organisational support, ,Relevance, Similar foundational knowledge, Consistent briefing &de-briefing | Improved perceptions of teamwork and Role clarity. Average time to place patients on ECMO decreased. Nosocomial infection rate was below upper control limits for 7/8 months. |  |
|  |  |  |  |  |  |  |  |  |  |
|  |  |  |  |  |  |  |  |  |  |
|  |  |  |  |  |  |  |  |  |  |
| **Author (Year)** | **Brief name of study** | **Location** | **Team descriptor** | **Intervention details** | **Study methodology** | **Teamwork, Mechanisms described** | **Contextual factors** | **Findings/Outcomes** |  |
| **Nakayama et al., (2010)** | High quality surgical service line | Tertiary referral centre | Hospital administration, nurses, central supplies, pharmacy, materials management, paediatric specialty, surgeons, anaestheologists, nurse leaders | Quality improvement | QI methodology |  | Necessary KSAs, Performing procedures in OR .Inter-disciplinary focus. Weekly walkthrough sessions by Physician with team .Performance data discussed monthly meeting. Team performance graded per procedure. VMS location | Improved team performance, communication, patient transfer and reduced problems with instruments, supplies, equipment and surgeon tardiness. |  |
|  |  |  |  |  |  |  |  |  |  |
|  |  |  |  |  |  |  |  |  |  |
|  |  |  |  |  |  |  |  |  |  |
|  |  |  |  |  |  |  |  |  |  |
| **Parker et al., (2010)** | Tracheostomy Management team performance | Tertiary Referral centre | Nurse consultants, speech pathologist, Dietitian, Social worker, medical officers, respiratory specialists | Development of an inter-disciplinary team | Mixed method enquiry incorporating qualitative and quantitative approaches | Clarity of role/ purpose, Mutual respect, support and value | Organisational acceptance of team. Rationalisation of services, Focus on patient outcomes, Regular team meetings and rounds. Prioritisation of patient goals, Effective communication, Monitoring strategies | Significant reduction in mean LOS |  |
|  |  |  |  |  |  |  |  |  |  |
|  |  |  |  |  |  |  |  |  |  |
|  |  |  |  |  |  |  |  |  |  |
|  |  |  |  |  |  |  |  |  |  |
| **Author (Year)** | **Brief name of study** | **Location** | **Team descriptor** | **Intervention details** | **Study methodology** | **Teamwork**  **Mechanisms described** | **Contextual factors** | **Findings/Outcomes** |  |
|  |  |  |  |  |  |  |  |  |  |
| **Patterson et al., (2012**) | Simulation based training using crew resource management in the ED | Tertiary Referral Centre | Physicians, Nurses, Respiratory Therapists, Paramedics, Patient Care Assistants and medical residents | Simulation based training using CRM principles | Interventional evaluation study | Motivating, engaging, empowering; Situational Awareness, shared understanding, shared mental models, Feeling knowledgeable and competent | Similar foundation Immediate video-assisted de-briefing, Practice of skills, In the present analysis, Reinforcement of desired behaviours, Professional hierarchies, Techniques to reduce medical error situational awareness, Video recordings Training in assertion & voicing concerns, ED leadership commitment, Continuous training | Patient Safety events reduced from 2-3 per year to none in 1000 days |  |
|  |  |  |  |  |  |  |  |  |  |
|  |  |  |  |  |  |  |  |  |  |
|  |  |  |  |  |  |  |  |  |  |
|  |  |  |  |  |  |  |  |  |  |
| **Riblet et al., (2014)** | Improvement in glioma care | Cancer Centre | Physicians, physician assistant, nursing staff, social workers, neuro-oncologist, neuro-surgeons, radiation oncologists, care mangers and cancer centre leadership | QI interventions | QI methodology | Motivating, satisfying, empowering, Sharing responsibility/ Collective responsibility, Connectedness, camaraderie, group,identity | Leadership &clinical involvement, Use of process maps, Trained QI coach, Communication systems, Awareness of organisational goals, Process for feedback, Stream-lined process, Performance measurement. | Improvement in use of 10 practice measures from 63% pre intervention to 85% post intervention in 96 consecutive patients. |  |
|  |  |  |  |  |  |  |  |  |  |
| **Author (Year)** | **Brief name of study** | **Location** | **Team descriptor** | **Intervention details** | **Study methodology** | **Teamwork**  **Mechanisms described** | **Contextual factors** | **Findings/Outcomes** |  |
| **Rosen et al., (2009)** | Implementation of family centred MDTs | In patient adolescent ward in Tertiary Referral Centre | Teaching and admitting Attendants, senior resident, intern, 4^th^ and 3^rd^ yea medical students, QI staff, nurse, social worker and pharmacist | Implementation of family centred MDT rounds | Quasi-experimental design | Motivating, satisfying, engaging. Mutual support, respect and value, Sharing responsibility, Feeling knowledgeable and competent. Connectedness, team identity,Sense of empowerment, confidence | Enablers and barriers described were relevant to MDT rounds as opposed to specific to teamwork. | No significant difference reported in family satisfaction between family centred rounds and conventional rounds. Better understanding of patient plans perceived by staff. |  |
|  |  |  |  |  |  |  |  |  |  |
|  |  |  |  |  |  |  |  |  |  |
| **Schmutz et al., (2015)** | Medical Emergency team training | Medical Emergency Dept & Paediatric ICU | Medical Emergency team- Nurses, residents, senior physicians | High fidelity in situ simulation | Hypothesis driven observational study | Situational awareness, shared understanding and shared mental model | Task distribution, Provision of information without request, Shared understanding, Closed loop communication, Task distractions, Personal biases and perspectives | Negative relationship between Provision of information without request led to negative clinical performance particularly in teams with an experienced leader. |  |
|  |  |  |  |  |  |  |  |  |  |
|  |  |  |  |  |  |  |  |  |  |
|  |  |  |  |  |  |  |  |  |  |
| **Author (Year)** | **Brief name of study** | **Location** | **Team descriptor** | **Intervention details** | **Study methodology** | **Teamwork**  **Mechanisms described** | **Contextual factors** | **Findings/Outcomes** |  |
|  |  |  |  |  |  |  |  |  |  |
| **Shea Lewis,(2009)** | Risk reduction programme, crew resource management | Community hospital obstetric dept. | Obstetricians, attending physicians, Midwife, registered nurses, and Team performance improvement | Implementation of specific communication tools and behaviours | Descriptive narrative | Situational awareness, Collaborative effort | Briefings and de-briefings. Education for all staff, Inter-disciplinary co-operation and on- going mentoring | Better patient outcomes as well as improved patient and staff satisfaction. |  |
| **Thomas and Galla, (2013)** | Team training TeamStepps | Community hospital, acute care facility | Teams trained in cohorts representing their work teams. no other descriptor | Implementation of TeamStepps | Narrative study | Motivating, Satisfying, Engaging, Empowering | Hospital leadership, supported &aligned with organisational strategy. Communication plan and process monitoring. Decisions communicated to all staff, Leadership rounds, Annual poster presentation, Competencies reviewed annually, Standardised process, Planned re-dosing, Physician leadership on all units, Trainers selected. | Significant improvement. System wide results, Reduction of nosocomial infections, falls, improvement in process measures and decrease in adverse outcomes, birth trauma and return to the OR. |  |
|  |  |  |  |  |  | |  |  |  |
|  |  |  |  |  |  |  |  |  |  |
|  |  |  |  |  |  |  |  |  |  |
|  |  |  |  |  |  |  |  |  |  |
| **Grey literature** | | | |  |  |  |  |  |  |
| **Author (Year)** | **Brief name of study** | **Location** | **Team descriptor** | **Intervention details** | **Study methodology** | **Teamwork**  **Mechanisms described** | **Contextual factors** | **Findings/Outcomes** |  |
| **Freeth et al., (2009)** | MOSES Promoting Patient Safety in obstetrics | 4 UK delivery suites purposively selected. to reflect range of demographics, Already participating in Safety study | Senior midwives, obstetricians, and obstetric anaesthetists | A 1-day simulation-based education course | Descriptive /Narrative analysis | Relationship building-Teamness, Respect for different roles within the team | Learning dependent on their foundational knowledge, Senior clinicians as facilitators, Links to practice, Supportive atmosphere, Performance discussed openly, Effective facilitation Entrenched hierarchies and inter-professional tension, Immediate application to practice, Realistic simulations, Opportunity for critical reflection, Lack of Mechanisms to support diffusion of learning, Management support | Participants reported acquiring new knowledge or insights, particularly concerning the role of communication and leadership in crisis situations, and they rehearsed unfamiliar skills |  |
|  |  |  |  |  |  |  |  |  |  |
|  |  |  |  |  |  |  |  |  |  |
|  |  |  |  |  |  |  |  |  |  |
|  |  |  |  |  |  |  |  |  |  |
|  |  |  |  |  |  |  |  |  |  |
|  |  |  |  |  |  |  |  |  |  |
|  |  |  |  |  |  |  |  |  |  |
| **O Leary et al.,**  **(2010)** | Improving Teamwork: Impact of Structured Interdisciplinary Rounds ((SIDR) | Tertiary Care Teaching hospital | Attending, Resident, Interns, Medical students, Nurses pharmacist, social worker, and case manager assigned to the unit | SIDR combined a structured format for communication and a forum for regular ID meetings | Randomised controlled study | Psychological safety, Nurses felt unable to approach Physicians. | Barrier to collaboration, Structured communication and daily plan of care. | IDR had a positive effect on nurses’ ratings of collaboration and teamwork on a medical teaching unit. |  |

**Hyperlink 2 Teamwork Mechanisms identified**

| **Teamwork Mechanisms identified Studies** | |
| --- | --- |
| Motivating, emotional engagement, empowerment | Hina Sayeda (2013); Patterson et al., (2012); Riblet et al., (2014); Thomas and Galla (2013); Parker et al., (2010); Rosen et al., (2009) |
| Clarity of role or purpose | Hina Sayeda, (2013); Parker et al., (2010); Nakayama et al., (2010); Ellaham et al., (2013); Deneckere et al., (2013) |
| Sense of shared accountability and responsibility | Hina Sayeda, 2013; Nakayama et al., (2010) |
| Mutual support, respect and value | Parker et al., (2010); Deneckere et al., (2013); Nakayama et al., (2010) |
| Situational awareness/monitoring, Shared understanding; shared mental models | Patterson et al., (2012); Donovan et al., (2016); Schmutz et al., (2015); Mayer et al., (2011); Rosen et al.,(2009); Cima et al., (2009) |
| Sharing responsibility, Collective responsibility | Hina Sayeda (2013); Riblet et al., (2014), Deneckere et al., (2013) ; Donovan et al, (2016); Schmutz et al., (2015); Rosen et al., (2009); Ellaham et al., (2013) |
| Self & team efficacy; Role contribution valued | Parker et al., (2010); Hina Sayeda (2013); Nakayama et al., (2010) Rosen et al., (2013) |
| Feeling knowledgeable/competent | Hina Sayeda (2013); Patterson et al., (2012); Deneckere et al., (2013); Donovan et al., (2016); Schmutz et al., (2015); Chiccocchi, Rabbatt and Lebel (2015); Figuero, Sepanski and Goldberg (2013) |
| Connectedness, Camaraderie, Team identity | Riblet et al.,(2014); Deneckere et al., (2013); Chiccochio, Rabbat and Lebel (2015) |
| Sense of empowerment/ confidence | Thomas and Galla (2013) ; Nakayama et al., (2010); Figuero, Sepanski and Goldberg (2013) |
